# A Theoretical Study of Organotin Binding in Aromatase

**DOI:** 10.3390/ijms24108954

**Published:** 2023-05-18

**Authors:** Shuming Cheng, Jing Yang

**Affiliations:** School of Chemical Engineering and Technology, Sun Yat-Sen University, Zhuhai 519082, China; chengshm3@mail2.sysu.edu.cn

**Keywords:** organotin, aromatase, molecular dynamics, binding energy, toxicology

## Abstract

The widely used organotin compounds are notorious for their acute toxicity. Experiments revealed that organotin might cause reproductive toxicity by reversibly inhibiting animal aromatase functioning. However, the inhibition mechanism is obscure, especially at the molecular level. Compared to experimental methods, theoretical approaches via computational simulations can help to gain a microscopic view of the mechanism. Here, in an initial attempt to uncover the mechanism, we combined molecular docking and classical molecular dynamics to investigate the binding between organotins and aromatase. The energetics analysis indicated that the van der Waals interaction is the primary driving force of binding the organic tail of organotin and the aromatase center. The hydrogen bond linkage trajectory analysis revealed that water plays a significant role in linking the ligand–water–protein triangle network. As an initial step in studying the mechanism of organotin inhibiting aromatase, this work provides an in-depth understanding of the binding mechanism of organotin. Further, our study will help to develop effective and environmentally friendly methods to treat animals that have already been contaminated by organotin, as well as sustainable solutions for organotin degradation.

## 1. Introduction

Organotins (OTs) are R_n_SnX_4-n_-type organometallic compounds; R represents organic substituents (such as methyl and butyl), and X represents non-carbon substituents (such as hydroxyl and halogens). Organotins are widely used in industry, agriculture, and marine transport as pesticides, wood preservatives, fungicides, catalysts for the plastic industry, and biocides for antifouling paint [1]. However, OTs, especially tributyltin (TBT) and triphenyltin (TPT), are well known as significant environmental endocrine disruptors, damaging the metabolism and neurological and reproductive systems of animals and humans. Although TBT has been banned for utilization in antifouling paint, the residual TBT in the marine environment still damages the ecosystem persistently due to the degradation difficulty and TBT bioaccumulation. Besides TBT as an additive in antifouling paint, TPT also threatens the environment and human health [2], which has not been considered thoroughly.

It is accepted that OTs can disrupt steroid metabolism by inhibiting cytochrome P450 aromatase (CYP19A1), one of the mechanisms of sexual aberration caused by OTs [2,3,4]. Cytochrome P450 aromatase is the only steroid synthase responsible for converting androgens to the corresponding estrogens. A typical example is the conversion of androstenedione (ASD) to estrone assisted by cytochrome P450. Therefore, aromatase is determinant in animals’ androgen/estrogen balance [5,6,7]. When OTs inhibit the aromatase activity, androgen accumulates, leading to a disorder of the steroid level.

Pioneers have investigated the impact of OTs on aromatase activity through in vitro experiments. It was found that TBT could reversibly and competitively inhibit human placental aromatase activity [8,9]. Tetrabutyltin, monobutyltin, and tri-, di-, and monooctyltins do not affect aromatase activity. The inhibition of aromatase activity by TPT has also been investigated [10], and the resulting inhibition constant Ki (0.53 μM) (IC_50_ = 1.5 μM, with 0.1 μM androstenedione as the substrate) was lower than that of TBT (50 μM), suggesting a stronger binding of TPT in aromatase. The inhibition effect of TPT on aromatase can be compensated by dithioerythritol, which also suggests a reversible inhibition mechanism.

Although experiments have revealed the interaction between aromatase and OTs at the macro level, the molecular mechanism remains unclear. An in-depth understanding of the molecular mechanisms may help to develop an effective method for treating OT-contaminated organisms and inspire new aromatase inhibitors. It is worth mentioning that OT metabolism in mammals is carried out by cytochrome P450 system enzymes, producing fewer toxic metabolites [11,12,13,14]. Whether aromatase is relevant to OT metabolism is still being determined. Because aromatase belongs to the cytochrome P450 family, we rationalize that understanding how aromatase degrades OTs may enlighten us on the mechanism of P450 degrading OTs in general. Therefore, the study of the OT binding in aromatase could help understand the OT metabolism mechanism.

Several studies have used computational methods, such as molecular docking and molecular dynamics simulation, to study the interaction between ligands and aromatase, assisting the design of aromatase inhibitors [7,15,16,17]. For example, Cevik et al. synthesized novel benzimidazole–oxadiazole derivatives as aromatase inhibitors and analyzed the interaction between inhibitors and active-site residues. Hydrophobic interaction is the main interaction type, including π–sulfur, π–π, and π–alkyl interactions, while conventional and π–donor hydrogen bonds have also been reported [15]. Zhang et al. investigated aromatase inhibition by glyphosate with molecular docking and molecular dynamics. Glyphosate could bind with an allosteric site in the access channel of the substrate and form hydrogen bonds with Asp-309, which is essential for substrate binding and the catalysis reaction [17]. 

In this work, we performed a detailed computational study to unveil the OT binding mechanism in aromatase. We applied molecular docking and classical molecular dynamics (MD) to investigate the binding of triphenyltin hydroxide (TPTOH) and tributyltin hydroxide (TBTOH) with human placental aromatase (CYP19A1). Combining binding free energy calculation with molecular mechanics/Poisson–Boltzmann surface area (MM/PBSA) method and quantum chemistry calculation, we found that the van der Waals (vdW) interaction is the primary driving force of the binding between the organic tail of organotin and the aromatase center. Furthermore, the trajectory analysis revealed that water plays a significant role in linking the ligand–water–protein triangle network, stabilizing the binding mode of OTs, and may assist proton transmission in the catalysis reaction of OT degradation. Our work provides insights into how OTs inhibit aromatase activity and contributes to understanding the OT degradation mechanism.

## 2. Results and Discussion

### 2.1. Molecular Docking and Clustering

**Figure 1:**
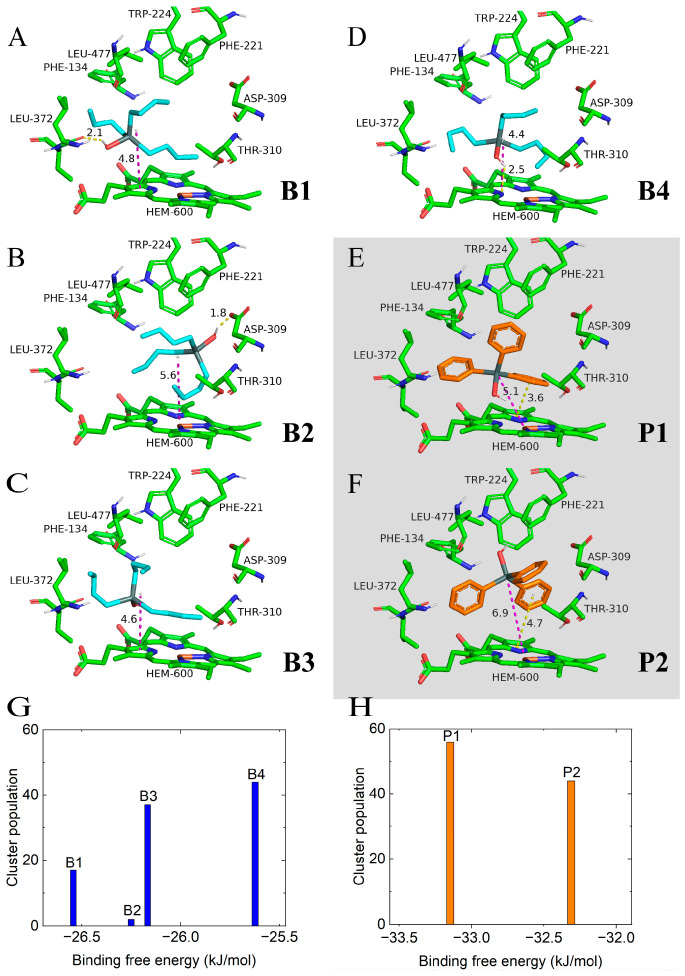
The representative binding conformations with the lowest binding free energy in clusters obtained through molecular docking and the number of conformations in each cluster. (**A**–**D**) TBTOH binding conformations in clusters **B1** to **B4**. The distances of TBTOH’s center-of-mass (C.M.) to heme plane are presented as purple dashed lines with the corresponding distance value. The hydrogen bonds (yellow dashed lines) and donor–acceptor (X⋯H, X = O or N) distances are shown (Å). (**E**,**F**) TPTOH binding conformations in cluster **P1** and cluster **P2**. The π–π interactions are presented as yellow dashes, and the Sn–Fe distances are presented as purple dashed lines with the corresponding distance values (Å). (**G**) The cluster populations of clusters **B1** to **B4**. (**H**) The cluster populations of cluster **P1** and cluster **P2**. The binding free energy of representative conformations of each cluster is shown.

To reveal the binding sites of organotins, TPTOH and TBTOH were docked into the active pocket of CYP19A1 using the Lamarckian genetic algorithm implemented in AutoDock 4.2.6. The resulting binding conformations were clustered with the internal clustering method in AutoDock using a 2 Å cutoff of the root mean square deviation (RMSD) (Figure 1). The binding free energy of ligand to protein was estimated with the AutoDock 4.2 force field. We found four clusters for TBTOH numbered **B1** to **B4** (Figure 1A–D) and two for TPTOH numbered **P1** and **P2** (Figure 1E,F). The conformation with the lowest binding free energy (−26.56 kJ/mol) was found in cluster **B1** (Figure 1A). **B2** contains the lowest number of conformations among the four clusters (Figure 1B). We found a common feature within **B1**, **B3**, and **B4**, where the butyl groups of TBTOH are more extended and lie closer to HEM than that of **B2**. Due to TBTOH being randomly oriented in the pocket and the long tail tending to extend in the space, the hydroxyl group is always freely exposed to the surroundings, resulting in hydrogen bonding with other residues. In clusters **B1**, **B2**, and **B4**, we labeled the bond distance between the hydroxyl of TBTOH and LEU-372, ASP-309, and HEM, respectively. The bond lengths vary within the hydrogen bond range from 1.8 Å to 2.5 Å. As for the TPTOH clusters, the conformation with the lowest binding free energy (−33.15 kcal/mol) was found in cluster **P1**. TPTOH in cluster **P1** stays closer to HEM, with the Sn–Fe distance shorter by 1.8 Å, and the three phenyl groups orient toward the hydrophobic residues (PHE-221, TRP-224, and LEU-477) around the opening of CYP19A1. Meanwhile, in cluster **P2**, the phenyl rings orient toward HEM. These phenomena indicate that the phenyl groups of TPTOH tend to form a π–π stacking interaction with HEM in cluster **P1**, while these rings form a T-shaped π–π interaction in cluster **P2**. Hydrogen bonding is rarely found in the TPTOH complex because the bulky phenyl groups drive the hydroxyl group away from the surroundings. 

### 2.2. Molecular Dynamics Simulation of Binding Modes

To uncover the binding modes of OTs, classical MD simulations were performed for TBTOH and TPTOH in complexes with CYP19A1. The initial structures were picked from the docking procedure: **B1** for the TBTOH complex and **P1** for the TPTOH complex. MD simulations were first performed for 30 ns; then, the trajectories were extended to 100 ns to evaluate the stability of the ligand–protein complexes. After the MD simulations, the RMSD of the CYP19A1-OT complexes and OTs were calculated on the MD trajectory. The backbone of the enzyme in each frame was aligned to the first frame to remove the effect of translation and rotation at the beginning of the RMSD calculations. During the production runs, the RMSD of the CYP19A1-TBTOH and CYP19A1-TPTOH complexes reached a stable state after 3 ns, with a root mean square error (RMSE) value lower than 0.15 Å (Table 1, Figure S2), indicating the stability of the CYP19A1-OT complexes. The rather noticeable fluctuation of the RMSD of TBTOH and TPTOH (RMSE for TBTOH: 0.376 Å; for TPTOH: 0.278 Å) indicated that the interaction between aromatase and OTs is not very strong, leaving OTs with relatively high freedom of movement. Due to the flexibility of butyl groups, TBTOH embraces a relatively large conformational change. 

**Figure 2:**
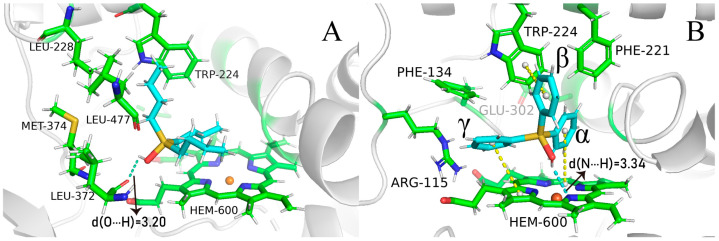
The binding modes of TBTOH (**A**) and TPTOH (**B**) with aromatase. The hydrogen bond s is presented with cyan dashed lines with the corresponding donor–acceptor distances (Å). The π–π interactions among TPTOH, TRP-224, and HEM are presented by yellow dashes. The three phenyl rings in TPTOH are labeled as α, β, and γ.

On the other hand, TPTOH contains rigid phenyl groups, leading to a smaller RMSE value. The RMSD curve of TPTOH has two stages: before 30 ns, the RMSD and its fluctuation slightly decreased, indicating a relatively stable interaction between CYP19A1 and TPTOH; after 30 ns, the RMSD and RMSD fluctuation increased, indicating a more flexible binding mode of TPTOH. We applied the clustering technique to the reduced trajectory of 3–30 ns and 30–100 ns at 100 ps sampling intervals, respectively. A single-linkage algorithm was used with a cutoff of 2.0 Å. Only one cluster was found for each trajectory fragment for the TBTOH and TPTOH complexes. For TBTOH, the binding conformations of the two trajectory fragments are almost the same. For TPTOH, despite the slight discrepancy, the types of interaction in the binding site remain the same (the 3–30 ns binding modes are shown in Figure 2, and the 30–70 ns binding modes are shown in Figure S3). In the following discussions, we applied detailed analysis to the 3–30 ns trajectory to gain microscopic insights for the OTs binding. 

We reapplied the clustering on the 3–30 ns trajectory at 10 ps sampling intervals, using a smaller cutoff of 0.99 Å for TBTOH and 0.97 Å for TPTOH. As a result, we found 17 clusters for TBTOH and 11 clusters for TPTOH (see Table S1 for the distribution). The stable binding modes for TBTOH and TPTOH are shown in Figure 2A,B (see Figure S4 for all the representative binding modes). Both cases share similar features: (1) The hydrophobic part of OTs governs the binding modes; (2) the TRP-224 from CYP19A1 forms a strong vdW interaction with TBTOH and TPTOH. Such a hydrophobic pocket formation of both OTs is related to the “like dissolves like” concept, which indicates that CYP19A1 tends to adapt the morphology of OT molecules to anchor the structure. Therefore, TRP-224 could be essential in stabilizing the binding between OTs and aromatase.

For the TBTOH complex, two butyl groups lie close to HEM, and the third one orients vertically and fits into the hydrophobic pocket formed by PHE-134, LEU-477, LEU-228, and TRP-224. Such a configuration leaves space for the hydroxyl group to interact with the carbonyl group on LEU-372, forming a hydrogen bond (Figure 2A). 

**Figure 3:**
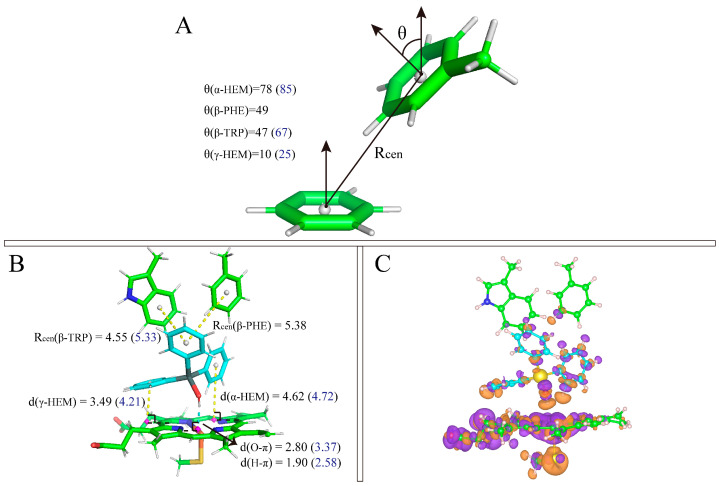
The π-π interaction dihedrals and distances of the TPTOH binding. (**A**) The definition of R_cen_ and dihedral θ. The dihedral between phenyl planes of the TPTOH and aromatic residues in the DFT optimization structure are shown (°), with the corresponding average values from the MD simulation in the bracket. (**B**) The R_cen_ values on the DFT structure and MD trajectory (in the bracket). The unit is Å. (**C**) Charge density difference of TPTOH binding calculated with ∆*ρ* = *ρ*(protein + TPTOH) − *ρ*(protein) − *ρ*(TPTOH). The isosurfaces of the value ±0.001 are shown. Purple means electron accumulation, and orange indicates electron density loss.

**Figure 4:**
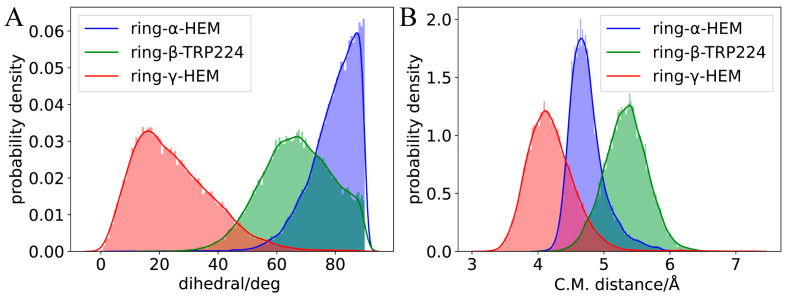
The distribution of π–π interaction dihedrals and distances. (**A**) The distribution of dihedrals (θ) between TPTOH phenyls and the aromatic rings of HEM or TRP-224. (**B**) The centroid distance (R_cen_) distribution between π–π interaction-related rings.

For TPTOH, the phenyl rings of TPTOH tend to interact with hydrophobic residues, especially PHE-134, PHE-221, and TRP-224. To identify the potential π-π interactions, we calculated the centroid distance of the aromatic ring (R_cen_), the distance from the centroid of the TPTOH phenyl ring perpendicular to the HEM plane (d), and the acute angle between two aromatic ring planes (θ), as defined in Figure 3A,B. For convenience, we labeled the three phenyl rings in TPTOH as α, β, and γ (Figure 2B). Due to the steric effect, the three rings are orthogonal to each other and tend to interact with HEM via a T-shaped π–π interaction to stabilize the binding mode. As a result, the distance between the ring-α and HEM (d(α-HEM)) increases to a final value of 4.72 Å, and the dihedral angle becomes 85° (Figure 4 and Figure S5). In addition, ring-β forms a T-shaped π–π interaction with TRP-224, where the R_cen_ of ring-β to TRP-224 is 5.33 Å, and the average dihedral angle is 67°. π–π stacking was also recognized between ring-γ and HEM as the ring lies parallel to the HEM plane. The distance between ring-γ and HEM (d(γ-HEM)) is around 4.21 Å, and the average dihedral angle is 25°. These data are consistent with previous findings [18]. 

During the simulation of TPTOH interacting with aromatase, we observed an interesting behavior: ring-α and ring-γ move by swinging against the HEM plane. In contrast, ring-γ is more mobile (Figure 4). This dramatic motion of ring-γ may be due to the interaction that HEM with ring-γ is weaker than with ring-α. In the meantime, the ring swing changes the orbital interaction between the phenyl rings and HEM, which might cause a charge fluctuation and then regulate the chemical reactivity of HEM. Since HEM is critical in catalytic reactions, the charge fluctuation might function as an activating motion for further enzymatic catalysis.

Substituting the hydroxyl group in OTs may alter the repulsion of the hydrophobic groups [18]. We identified the hydrogen bonding between OTs and CYP19A1 under a threshold where the X⋯H distance (X represents the O atom or N atom of nearby residues) is below 2.5 Å, and the hydrogen bond angle is greater than 120° [19,20]. Under this threshold, we identified one type of hydrogen bond between the hydroxyl group and the oxygen atom on LEU-372 (Figure 2A). The bond-forming probability is 0.20 between the hydroxyl group of TBTOH and LEU-372, and the average O⋯H distance is 3.2 Å; the average H bond angle is 134° (see SI for the definition of bond-forming probability). As for TPTOH, the hydroxyl group binds with one of the nitrogen atoms on HEM, forming OH⋯N, with a bond-forming probability of 0.08. These phenomena show that the hydrogen bonding between OTs and CYP19A1 is relatively weak, indicating that the polar substitution contributes little to the OT binding.

To obtain more reliable results about the π-π interaction between TPTOH and CYP19A1, we performed density functional theory (DFT) calculation on a cluster model consisting of TPTOH, HEM, PHE-221, TRP-224 and CYS-437. Geometry optimization was first performed, followed by the binding energy calculation. As shown in Figure 3A,B, ring-α forms T-shaped π-π interaction with HEM, while ring-γ and HEM form an offset stacked conformation. Ring-β is in an intermediate conformation with TRP-224 and PHE-221. All of the π-π interaction distances are in the range of typical π-π interactions [18,21]. Therefore, we can ensure the existence of a π-π interaction in the CYP19A1-TPTOH complex. The calculated binding energy is −116.108 kJ/mol, indicating the π-π interactions are strong and significantly contribute to the TPTOH binding. Applying symmetry-adapted perturbation theory (SAPT) calculation (Table S2), we found that dispersion dominates the π-π interaction between TPTOH and PHE-221 or TRP-224. We notice that in DFT optimized structure, the hydroxyl of TPTOH tends to form a hydrogen bond with HEM. Figure 3C shows a noticeable charge transfer during the TPTOH binding, indicating the electrostatic and induction contributions are much larger in the π-π interaction between TPTOH and HEM, which is a stronger orbital interaction. 

### 2.3. Binding Energy Analysis

To gain a quantitative understanding of the binding, we performed MM/PBSA calculations on the frames extracted from 3–30 ns trajectory at 100 ps intervals to analyze the energetics during the molecular binding process (Table 2). Compared to the experimental value [9,10], the binding free energy calculated using the MM/PBSA method was significantly overestimated. However, the calculation reproduced the tendency for TBTOH to possess a lower |∆*G_bind_*| than that of TPTOH (Table 3).

Consistent with the previous binding configuration, the non-covalent interaction is dominant in the TBTOH and TPTOH complexes. The gas phase interaction energy between OTs and CYP15A1, Δ*E_gas_*, can be represented by the summation of the van der Waals (vdW) interaction (Δ*E_vdW_*) and the Coulomb interaction (Δ*E_Coul_*),

(1) $$\Delta E_{gas}=\Delta E_{vdw}+\Delta E_{Coul}.$$

The Δ*E_vdW_* contribution to the TPTOH complex is greater than that to the TBTOH complex, as TPTOH forms a T-shaped π–π interaction with the hydrophobic residues. The larger Coulomb contribution to the TBTOH complex than that to the TPTOH complex indicates that the polar hydroxyl group is more involved in the TBTOH complex. These results are consistent with the hydrogen-bond-forming probability, as illustrated in the previous section. Besides the van der Waals interaction, the gas phase entropy and polar solvation free energy contribute to the difference between the ∆*G_bind_* of TBTOH and TPTOH (Equation (7)). The long chains of TBTOH are flexible and require more residues to hold, resulting in larger binding entropy. 

**Figure 5:**
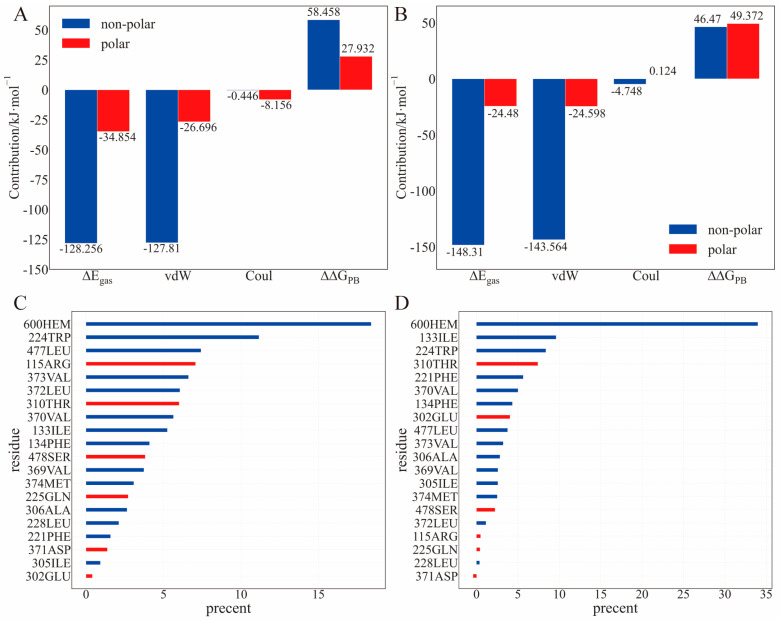
The decomposition of the MM/PBSA binding enthalpy and its components. (**A**,**B**) The binding site residues are divided into two groups: non-polar and polar residues, and the contributions of the two groups to the main components of ∆*G_bind_* are represented by the bars. (**A**) TBTOH. (**B**) TPTOH. (**C**,**D**) The contribution of each binding site residue to Δ*E_gas_*: red, non-polar; blue, polar. (**C**) TBTOH. (**D**) TPTOH.

Regarding the residue components in the binding energy, the non-polar residues contribute the most to the binding enthalpy, mainly via the vdW interaction (Figure 5A,B). Some common residues involving TBTOH and TPTOH binding (contribution > 8%) are ILE-133, TRP-224, PHE-134, and VAL-370 (Figure 5C,D). Note that LEU-372 and LEU-477 are hydrophobic but accidentally have high ΔΔ*G_PB_* in the TBTOH binding. The reason is that the C=O group in LEU-372 and the N-H group in LEU-477 tend to expose their polar end to bind with TBTOH (Figure S6E,F). Indeed, LEU-372 forms a hydrogen bond with TBTOH, leading to a stable binding conformation. However, the high ΔΔ*G_PB_* contribution may indicate a less favored binding in an aqueous environment.

With the above energetics analysis, we conclude the following: (1) OT binding with aromatase is dominated by the vdW interaction, especially with hydrophobic residues. The π–π interaction is essential in stabilizing the TPTOH binding mode. (2) The entropy contribution cannot be ignored, preventing OTs from binding with aromatase. The difference in the binding entropy and polar solvation free energy is the reason why TBTOH has a lower ∆*G_bind_*. (3) The commonly shared residues in both the TBTOH and TPTOH complexes may stabilize OTs for further enzymatic catalytic reactions.

### 2.4. The Role of Interfacial Water Molecules in OT Binding

**Figure 6:**
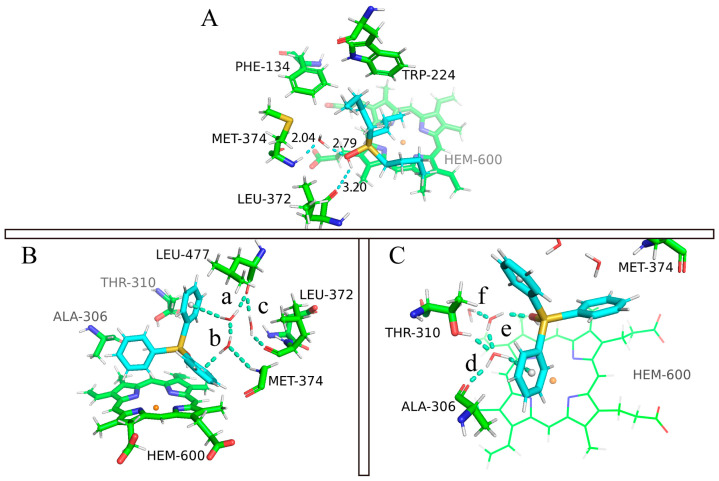
The ligand–water–protein triangle networks of TBTOH and TPTOH. The hydrogen bonds are presented as cyan dashed lines with the corresponding average donor–acceptor distance value. The geometrical centers of phenyls are presented as white spheres in the phenyl rings. (**A**) The TBTOH–water–MET-374 hydrogen bond bridge (Bridge 1) along with the hydrogen bond between TBTOH and LEU-372 is shown. (**B**) Network 1 of TPTOH. The three waters in Network 1 are labeled as a, b, and c. (**C**) The stable pattern of Network 2 formed by TPTOH, water, THR-310, and ALA-306. The three waters in Network 2 are labeled as d, e, and f.

In the MM/PBSA calculation, the solvent environment was modeled as a polarizable continuum, the MM/PBSA binding free energy does not include the explicit interactions between ligand and water. However, we know that the role of water molecules could be critical for various biological processes, including ligand binding. Here, we carefully analyzed the MD trajectory and observed a ligand–water–protein interaction network in both cases (Figure 6). Unlike the gas phase interaction, the hydrophilic parts of the surrounding residues become more involved in OT binding. For example, MET-374 and LEU-372 show closer binding in the TBTOH and TPTOH complexes than in gas phase systems. Polar residues such as THR-310 and ALA-306 interact with TPTOH frequently to form hydrogen networks. These networks play a crucial role in stabilizing the binding modes. 

In the TBTOH complex, we found a very stable hydrogen bond bridge mediated by a water molecule between the oxygen from the -OH of TBTOH and the hydrogen of the -NH on MET-374, which can be expressed as (Sn)-HO⋯H_2_O⋯HN-(MET-374) (Figure 6A). The short hydrogen bond donor–accepter distances (d(O_w_⋯HN_MET_-) = 2.04 Å, d(O_w_-N_MET_) = 3.01 Å and d(-HO_TBT_⋯O_w_) = 2.79 Å, O_w_ is the oxygen atom of water) indicate a strong interaction within the bridge. Therefore, water molecules in the environment are essential to the binding, assisting the ligand binding via a hydrogen bond bridge. 

The binding condition in the TPTOH complex is intricate when environmental water is involved. The reason is that the delocalized π-bonding in phenyl rings can also work as a hydrogen bond donor to form hydrogen bonds with surrounding water molecules. Different from the TBTOH system, two hydrogen bond networks form around TPTOH, which are mediated by three water molecules (Figure 6B,C). In Network 1 (Figure 6B), the interfacial water molecules are confined in the space surrounded by ring-β, ring-γ, MET-374, LEU-372, and LEU-477. In this region, the phenyl rings, O atoms from the water, and carbonyls of LEU-372 and LEU-477 act as hydrogen bond donors, while amides of MET-374 and water hydrogens act as acceptors. Such a network consists of two hydrogen bond bridges. Water-a links ring-β and LEU-477, forming one side of Bridge 1. Water-b connects ring-γ and MET-374, interlinking with water-a and completing Bridge 1. The other bridge joins the two residues via the carbonyl group on LEU-477 and LEU-372. Bridge 1 forms an interlinking network, indicating a geometrically stable structure during the dynamical process, since we also observed frequent inter-exchanges between water-b and water-c. In Network 2 (Figure 6C), two water molecules mediate the network between TPTOH hydroxyl oxygen, THR-310(-OH), ALA-306(-NH), and ring-α. Water-d connects ALA-306 and ring-*α*. Water-e joins water-d and the -OH of TPTOH to form a hydrogen bond network. In the meantime, water-d also connects to the -OH of THR-310. Similar to Network 1, the water molecules of e and f also undergo inter-exchanges during the simulation.

**Figure 7:**
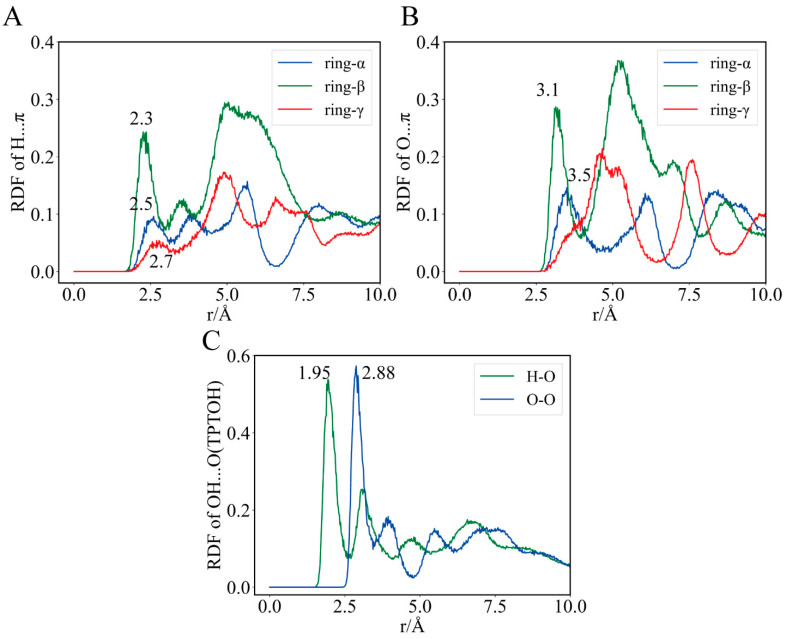
The radial distribution function (RDF) of the hydrogen bond donor–receptor pair between TPTOH and waters. The location of significant peaks is labeled. (**A**) RDF of phenyl center and water H atom pairs. (**B**) Phenyl center and water O pairs. (**C**) TPTOH hydroxyl O and water H pairs, and TPTOH hydroxyl O and water O pairs.

To identify the strength and stability of the hydrogen bond network, we performed radial distribution function (RDF) analysis for all the water molecules around TPTOH. In particular, we considered three interaction systems, HOH⋯π, H_2_O⋯π, and H_2_O⋯HO. Here, π indicates the π character of the phenyl group in TPTOH. The radial distances are those of the H or O atoms in the water to the geometrical of the phenyl rings (Figure 7A,B). The RDF of H_2_O⋯HO- represents the atom species within the water interacting with the hydroxyl of TPTOH (Figure 7C). 

The first peak of the RDF of the H⋯π curve occurs at the distance of 2.3 Å for ring-β, followed by the peak at 2.5 Å for ring-α, and 2.7 Å for ring-γ. All these peak distances are within the value obtained from the quantum chemical calculation (d(H⋯π) = 2.7191 Å) [22], indicating a stable hydrogen-bonding character. The peak height decreases following the order of ring-β, ring-α, and ring-γ. This means the surrounding water molecules around ring-β are more confined than the other two rings, which could explain the phenomenon whereby ring-β was always fixed to its position during the simulation. Hydrogen bonds around ring-β are also more vital than other rings. The lower first peak of the H⋯π RDF for ring-α and ring-γ reveals different binding mechanisms. Ring-γ is mobile and cannot form a rigid network with the surrounding water molecules, which leads to the lowest first peak of the RDF. The liquid-like nature of the RDF also indicates that the water molecules are more flexible near ring-γ. For ring-α, hydrogen bonding is not as strong as that of ring-β, as the ring-α movements occur majorly due to the T-shaped π–π interaction with HEM, partially correlating with the hydrogen bonding via the surrounding waters. 

The RDF of O⋯π presents a feature similar to that of H⋯π. Only the first peak of ring-β falls below 3.3 Å; the quantum chemical calculation suggested that d(O⋯π) is 3.3085 Å and experimental d(O⋯π) is 3.411 Å [22]. Indeed, the hydrogen bond network around ring-β is stronger than that of the other two rings. The slightly underestimated O⋯π peak position for ring-*α* and ring-γ may be due to the system error caused by force field parameters. The sharpest peaks can be observed in the RDF for hydrogen bonding between the -OH of TPTOH and water molecules amongst the three types of RDFs. Furthermore, water molecules are mostly confined around the -OH group, indicating a robust hydrogen bond network.

According to the analysis above, we conclude that interfacial water molecules are crucial to OT binding with aromatase. Interfacial water molecules dominate the hydrogen bond network formation and stabilize the OT–aromatase binding structure. Additionally, the complexity of the network may lead to high binding affinity. The TBTOH–aromatase complex contains a single hydrogen bond bridge, whereas two networks with multiple bridges are formed in the TPTOH complex. The short-range interaction energy of the TPTOH–aromatase (−248.152 kJ/mol) is lower than that of the TBTOH–aromatase (−243.990 kJ/mol), which also reinforces the above point of view (Table S3).

The analysis of the hydrogen bond trajectory revealed that water plays an essential role in linking the ligand–water–protein triangular network, which promotes the stability of the OT binding configuration. In addition, water in the triangular network may participate in catalytic reactions to degrade OTs and work as a proton transfer channel [23,24].

By comparing and contrasting the binding of TBTOH and TPTOH to aromatase, we found that the common driving force for the two binding complexes is vdW interactions, which mainly rely on the interaction with non-polar residues to stabilize the binding configuration. The reason is that OTs primarily comprise hydrophobic hydrocarbon groups and aromatic rings. The difference is that the binding of TPTOH is more potent than that of TBTOH because the butyl group of TBTOH is flexible, while the benzene ring of TPTOH is rigid. The flexible structure makes the binding entropy of TBTOH larger, which is not conducive to binding; in addition, the phenyl group of TPTOH can provide π-electrons to form π-type hydrogen bonds with water molecules, resulting in a triangular network. The phenyl group also forms π–π interactions with the aromatic groups of residues, which increase the tendency of TPTOH to bind strongly. All these results suggest that we need to appropriately reduce molecular flexibility to design P450 inhibitors, thereby reducing unfavorable binding entropy and using aromatic groups to enhance the interaction between inhibitors, proteins, and water. As for the enlightenment on enzyme catalysis, TBTOH has more obvious conformational changes than TPTOH, while TPTOH is relatively more tightly bound. The tighter binding confines the substrate near the top of HEM, which is conducive to the catalytic reaction progressing.

## 3. Methods and Materials

### 3.1. Model Preparation and Molecular Docking

The starting structure of CYP19A1 in complex with its natural substrate androstenedione (ASD) was obtained from the X-ray structure (PBD ID: 3EQM) [25]. The crystal waters, ASD and phosphate ions were removed, and the resulting enzyme coordinate was used for molecular docking. The stable forms of the corresponding organotins, such as TPTOH and TBTOH, in a neutral aqueous environment, were built in Avogadro [26]. Then, molecular docking for each ligand was performed with AutoDock4.2.1 with 100 docking runs [27]. Note that the Lennard–Jones parameters of Sn atoms are not provided in AutoDock4.2.1. We extracted those parameters from UFF force field directly [28]. The lowest energy conformations in complex with CYP19A1 were selected as the input for the MD simulation.

### 3.2. System Construction and Topology Preparation 

The protonation states of the CYP19A1 residues were assigned using the H^++^ website [29,30,31] assuming a pH of 7.0 with all other default settings, and the missing hydrogens of all standard residues were added. The ASP309 residue was in a deprotonated state. Although protonated ASP309 plays a key role in substrate binding and catalytic reactions [23,24], our molecular docking and molecular dynamics modeling revealed that the binding mode of organotin is not conducive to the formation of hydrogen bonds with protonated ASP309. The hydrogen bonds are mainly electrostatic interactions, while the main driving force for OT binding is vdW force. Therefore, protonated ASP309 was not necessary in our case. The Cys437 residue was manually modified into its deprotonated form to coordinate with the heme iron, and missing hydrogens of heme were added using Avogadro. Then, TPTOH or TBTOH with coordinates taken from the docking result was placed in the pre-processed enzyme. The obtained complex was solvated with TIP3P [32] water in a cubic box, and the minimum distance between the complex and the boundary was set to 10 Å. A total of 5 Cl^−^ ions were added to neutralize the system along with additional 79 Na^+^ and 79 Cl^−^ ions to model a 150 mM NaCl solution. The final system contained CYP19A1, a ligand (TPTOH or TBTOH), 79 Na^+^ ions, 84 Cl^−^ ions, and about 26,000 water molecules. Aromatase is a membrane protein, and the membrane environment may affect substrate binding and catalysis by affecting substrate access and interactions between aromatase and membrane-anchored proteins [33]. Accurate modeling of the kinetics of organotin binding requires the enzyme to be embedded in phospholipid membranes, but this was outside the scope of our study. In addition, the membrane environment does not significantly affect the structural properties of aromatase [6], so the current model should be sufficient to reveal the interaction of aromatase with OTs.

The AMBER ff14SB [34] force field was employed for all standard residues, while the partial charges in the heme and its axial ligand Cys437 and the force field parameter of heme were obtained from Shahrokh et al. [35]. The Lennard–Jones parameters of Sn were adopted from Li et al. [36]. For the topology of the ligands, the generalized AMBER force field [37] (GAFF) form was applied. Geometry optimization and frequency analysis for TPTOH and TBTOH were performed in ORCA4.2.1 [38,39], using the B3LYP [40,41,42] functional and a mixing basis with LANL2DZ [43] for Fe and Sn, and 6-311G* [44] for other elements; then, the Hessian matrices obtained from the frequency analysis were used as the input for VFFDT [45]. Following this, the bond and angle parameters of TPTOH and TBTOH were derived using the VFFDT program. To obtain the partial charges of TPTOH and TBTOH, single-point calculations at the same level were carried out with the conductor-like polarizable continuum (CPCM) [46] solvation model (water solvent) on the optimized geometry; then, the restrained electrostatic potential [47] charges were calculated in Multiwfn [48]. The X-C-C-X (X = Sn, C, or H), H-C-C-H, and C-C-C-C dihedral parameters obtained from GAFF were applied to TPTOH and TBTOH in order to make sure that the atoms on the benzene rings remained on the plane and Sn was on the same plane during the MD simulation. It should be pointed out that the force field charges are fixed during the simulation.

### 3.3. Molecular Dynamics Simulations

GROMACS 2019.6 [49] was used for all the molecular mechanics (MM) simulations, including the minimization, equilibration, and production, using parameters derived from the process described above. The system was initially minimized to remove unfavorable contacts and interaction with the steepest descent algorithm, with a threshold of 500 kJ·mol^−1^·nm^−1^. The equilibration steps were run in the NVT ensemble at 310 K for 100 ps, followed by the NPT ensemble at 310 K and 1 bar for 500 ps. The time step for the NVT and NPT equilibria was 2 fs.

Subsequently, the MD production of the system was run in the NPT ensemble without any constraints for 100 ns. During the simulation, the coordinates and energy of the system were dumped every picosecond, resulting in a trajectory of 30,000 individual conformations. The particle mesh Ewald [50,51] algorithm was employed for all MM simulations to calculate the long-range electrostatic interactions, and dispersion correction was applied. The short-range electrostatic and van der Waals interaction cutoffs were set to 10.0 Å. Harmonic position constraints were employed in the equilibration steps for the solute (CYP19A1 and ligand), whereas hydrogen-heavy atom bond lengths were constrained with the LINCS algorithm. A velocity-rescaling [52] thermostat with a relaxation time of 0.1 ps was used to control the temperature in the equilibration steps, and the Berendsen barostat [53] with a relaxation time of 0.3 ps was employed to control the pressure. For the MD production run, the Nose–Hoover thermostat [54,55] with a time constant of 1.0 ps and the Parrinello–Rahman barostat with a time constant of 2.0 ps were used.

### 3.4. Binding Free Energy Calculations

Before the binding free energy calculation, all trajectory frames were aligned with the first frame, and the root mean square deviation (RMSD) calculation was carried out. The RMSD curve of the protein–ligand complex showed that the structure of CPY19A1-TPTOH was fully relaxed at 3 ns. Therefore, we chose 3–30 ns trajectories for the binding free energy calculation. In order to estimate the binding free energy between CYP19A1 and ligands, we extracted 271 snapshots from 3–30 ns trajectories using the uniform sampling method with a sampling interval of 100 ps intervals. The binding free energy was calculated as the average value on these snapshots using the molecular mechanics/Poisson–Boltzmann surface area (MM/PBSA) [56,57] method with the shell script gmx_mmpbsa [58]. The MM/PBSA binding free energy can be calculated using the equation below:

(2) $$\Delta G_{bind}=\Delta G_{gas}+\Delta G_{sol},$$

where Δ*G_gas_* is the gas phase contribution expressed as

(3) $$\Delta G_{gas}=\Delta H_{gas}-T\Delta S_{gas}\approx \Delta E_{gas}-T\Delta S_{gas},$$

where Δ*E_gas_* is the interaction energy between the protein and the ligand; Δ*S_gas_* is the entropy difference between the protein–ligand complex, separated protein, and ligand in the gas phase, which is calculated using the interaction entropy method [56]; Δ*G_sol_* is the solvation contribution of free energy, which can be decomposed into the polar and non-polar components,

(4) $$\Delta G_{sol}=\Delta \Delta G_{PB}+\Delta \Delta G_{SA},$$

where ΔΔ*G_PB_* is the difference between the polar solvation free energy of the complex and the separated protein–ligand pairs, which can be obtained by solving the Poisson–Boltzmann equation using the APBS program [59]; ΔΔ*G_SA_* is the non-polar contribution, estimated using the empirical solvent-accessible surface area. The residual contributions of these components were also estimated using the script mentioned above. The −*T*Δ*S_gas_* term was calculated using the equation below:

(5) $$-T\Delta S_{gas}=k_{B}Tlne^{\beta \Delta E_{pl}^{int}}=k_{B}Tln\left(\frac{1}{N}\overset{N}{\underset{i=1}{\sum }}e^{\beta \Delta E_{pl,i}^{int}}\right),$$

where *β* = 1/k_B_T, and the temperature was set to 298.15 K; $\Delta E_{pl,i}^{int}$ is the fluctuation of protein−ligand interaction energy around the average energy of each frame in the reduced trajectories:

(6) $$\Delta E_{pl,i}^{int}=E_{pl,i}^{int}-\langle E_{pl}^{int}\rangle =E_{pl,i}^{int}-\frac{1}{N}\overset{N}{\underset{j=1}{\sum }}E_{pl,j}^{int}.$$

The protein–ligand interaction energy $E_{pl,i}^{int}$ was calculated separately with GROMACS. 

After reaching convergence (Supplementary Figure S1), the −*T*Δ*S_gas_* term was added with the Δ*E_gas_* and Δ*G_sol_* term:

(7) $$\Delta G_{bind}=\Delta E_{gas}-T\Delta S_{gas}+\Delta G_{sol}.$$

### 3.5. Trajectory Analysis

We extracted 2701 frames of the protein–ligand complex along the 3–30 ns trajectory using the uniform sampling method, with a sampling interval of 10 ps. Then, we performed clustering using a single-linkage algorithm as implemented in the gmx cluster tool in the GROAMCS2019.6 package. Other analyses of the trajectory were performed with Python scripts using the MDtraj [60] package. VMD [61] and pyMOL (open-source edition) were utilized to visualize the molecules and trajectories. 

### 3.6. Quantum Chemistry Calculation for The π-π Interaction

The density functional theory (DFT) calculations on the cluster model (net charge of -2, spin multiplicity of 6) consisting of TPTOH, HEM, and the side chain of PHE-221, TRP-224 and CYS-437 were performed in ORCA4.2.1 [38,39], using the B3LYP functional [40,41,42] with Grimme’s D3BJ dispersion correction [62,63]. We used a mixing basis with LANL2DZ [43] for Fe and Sn and 6-31G* [64,65] for the remaining elements. The initial structure for geometry optimization was extracted from the frame shown in Figure 2B. The dangling bonds of the boundary atoms were capped with hydrogen. During optimization, the Cartesian coordinates of C, N, and O atoms in PHE-221, TRP-221, the porphyrin ring, and the propionate group of HEM were fixed at their initial positions, leaving other atoms free to move. The protein environment was mimicked with the CPCM implicit solvent model (ε = 4). Then, we calculated the binding energy of TPTOH at B3LYP-D3BJ/6-311G** [44,66] level (using the same basis set as the geometry optimization for Sn and Fe) with counterpoise correction [67] in the vacuum. The symmetry adapted perturbation theory calculations were performed at the sSAPT0 [68] level for the TPTOH-PHE-221 and TPTOH-TRP-224 pairs on their DFT-optimized structure in Psi4 version 1.4 [69]. In the calculation, we used the def2-TZVPP [70] basis set for Sn and jun-cc-pVDZ [71] for other atoms. 

## 4. Conclusions

In the present work, we investigated the molecular mechanism of OT binding with aromatase, mainly using molecular docking and molecular dynamics. We focused on the binding condition of two representative OT ligands (TBTOH and TPTOH) with aromatase. OTs tend to bind with the hydrophobic active pocket of aromatase. The main driving force of the binding is the vdW interaction between OTs and hydrophobic residues. The Coulomb interaction has a greater effect on the TBTOH complex than the TPTOH complex, whereas the π–π interaction is significant in the TPTOH complex. The binding free energy trend of OTs, |Δ*G_bind_*|(TBTOH) < |Δ*G_bind_*|(TPTOH), was reproduced through the MM/PBSA calculation. However, the larger binding entropy change in the TBTOH complex than in the TPTOH complex indicates the weaker binding affinity of TBTOH in the aqueous phase. We also found that the interfacial water molecules are crucial to the binding condition. The newly formed H⋯π bonding in the TPTOH complex leads to a more complex hydrogen bond network and further stabilizes the binding of TPTOH over TBTOH. By clearly understanding the binding condition of OTs with aromatase at the atomistic level, we could gain insights into how organotin inhibits the function of aromatase as a first step. Our research will assist the development of effective and environmentally friendly methods for organotin pollution, and the search for sustainable solutions for organotin degradation.

## Figures and Tables

**Table 1:** The average value and RMSE value of RMSD in 3–100 ns trajectory.

System	Average RMSD/Å	RMSE/Å
CYP19A1-TBTOH complex	2.072	0.136
CYP19A1-TPTOH complex	2.006	0.125
TBTOH	3.258	0.376
TPTOH	2.045	0.278

**Table 2:** The result of the MM/PBSA calculation. “vdW” represents the van der Waals contribution to ∆*G_bind_*. “Coul” represents the Coulomb contribution to ∆*G_bind_*. All energy values are in kJ/mol.

Ligand	∆*G_bind_*	Δ*E_gas_*		ΔΔ*G_PB_*	ΔΔ*G_SA_*	−*T*Δ*S_gas_*
		vdW	Coul			
TBTOH	−78.385	−184.022	−18.402	115.953	−21.718	29.804
TPTOH	−90.732	−195.024	−1.941	105.751	−21.482	21.963

**Table 3:** Binding free energy from the MM/PBSA calculation and the experiments.

System	$\Delta G_{bind}^{cal}\;(\mathrm{kJ}/\mathrm{mol})$	$\Delta G_{bind}^{exp}\;(\mathrm{kJ}/\mathrm{mol})$
TBTOH	−78.385	−25.52 ^1^
TPTOH	−90.732	−37.24 ^2^

^1^ Gerard M. Cooke et al. [9]. ^2^ Susan Lo et al. [10].

## Data Availability

Data will be made available on request.

## Supplementary Materials

The following supporting information can be downloaded at: https://www.mdpi.com/article/10.3390/ijms24108954/s1.
