# Supplementary material for: A Theoretical Study of Organotin Binding in Aromatase
Source: Int J Mol Sci. 2023 May 18;24(10):8954. doi: 10.3390/ijms24108954 (PMC10218834; doi:10.3390/ijms24108954)
Supplement: Supplementary file 1 [file ijms-24-08954-s001.zip › ijms-2345440-supplementary.pdf]

Supplementary material

A Theoretical Study of Organotin Binding in Aromatase

Shuming Cheng[1] and Jing Yang[1,*]

[1]School of Chemical Engineering and Technology, Sun Yat-sen University, Zhuhai Campus, China; yangjing25@mail.sysu.edu.cn
*Correspondence: yangjing25@mail.sysu.edu.cn

Supporting Information Content

Figure S1. Convergence of interaction entropy of TBTOH- and TPTOH-complex. 2

Figure S2. The RMSD of TBTOH-aromatase complex (A), TPTOH-aromatase complex (B), TBTOH (C) and TPTOH (D). 2

Figure S3. The binding modes of TBTOH (A) and TPTOH (B)(C) with aromatase in the last 70 ns trajectories. The hydrogen bonds are represented by cyan dash, and the donor-acceptor distances are labeled (Å). The π-π interactions between TPTOH and TRP-224, HEM are presented as yellow dashes. 2

Figure S4. The binding modes of TBTOH (A)(B) and TPTOH (C) with aromatase in the 3-30 ns trajectories. The hydrogen bonds are represented by cyan dash. The π-π interactions between TPTOH and TRP-224, HEM are presented as yellow dashes. (A) The dominant binding mode of TBTOH. (B) The unstable binding mode of TBTOH. C: The binding mode of TPTOH. 3

Figure S5. The π-π interaction distance (A) and dihedral (B) between TPTOH ring-α and HEM-600 as function of time during MD simulation. 4

Figure S6. The contribution of each binding sites residues to the main components of ΔG_{bind}: ΔE_{gas} (A-B), van der Waals contribution (C-D) and $\Delta\Delta G_{PB}$ (E-F). (A, C, E) TBTOH. (B, D, F) TPTOH. 5

Table S1. The clustering result of MD trajectory. The number of frames in each cluster was shown in the table below. The cluster 1 to 10 and 13 to 17 correspond to binding mode1 of TBTOH, and cluster 11 and 12 correspond to mode2. 6

Table S2. The total SAPT interaction energy between TPTOH and TRP-224 or PHE-221 and its electrostatics, exchange, induction and dispersion components (kJ/mol). 6

Table S3. The mean value of the short-range organotin-water interaction energy and its Coulomb and van der Waals components (kJ/mol). ΔE_{total}=ΔE_{total}+ΔE_{water}. ΔE_{water} is the sum of coulomb and van der Waals components of the short-range organotin-water interaction energy. 6

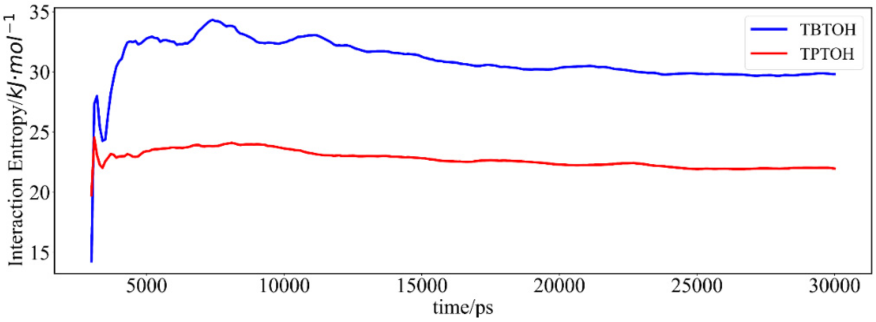

Figure S1. Convergence of interaction entropy of TBTOH- and TPTOH-complex.

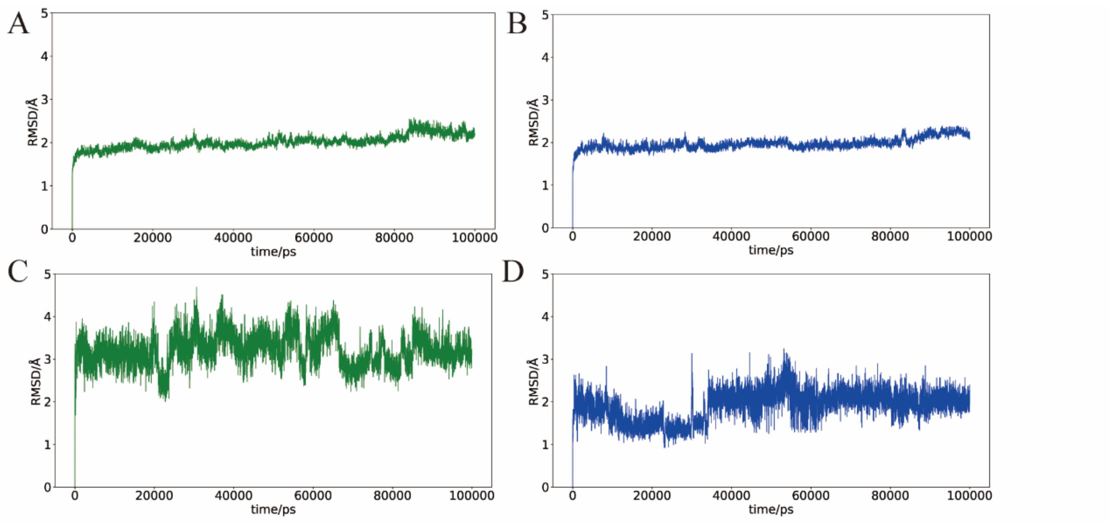

Figure S2. The RMSD of TBTOH-aromatase complex (A), TPTOH-aromatase complex (B), TBTOH (C) and TPTOH (D).

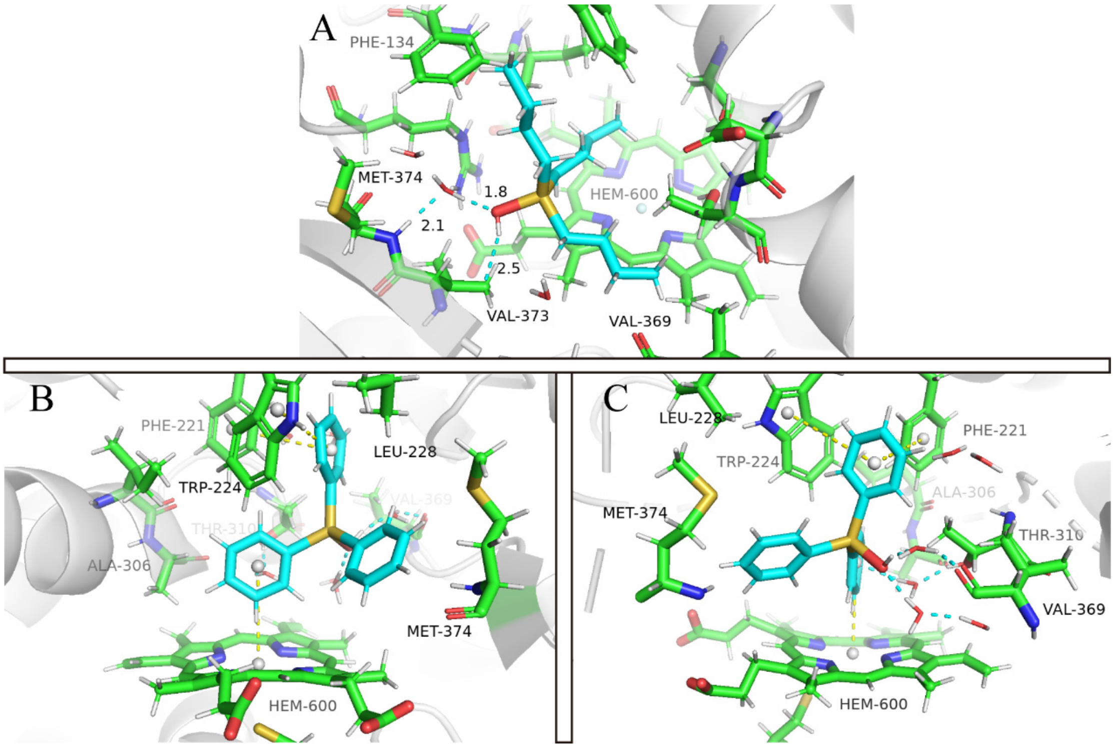

Figure S3. The binding modes of TBTOH (A) and TPTOH (B)(C) with aromatase in the last 70 ns

trajectories. The hydrogen bonds are represented by cyan dash, and the donor-acceptor distances are labeled (Å). The π-π interactions between TPTOH and TRP-224, HEM are presented as yellow dashes.

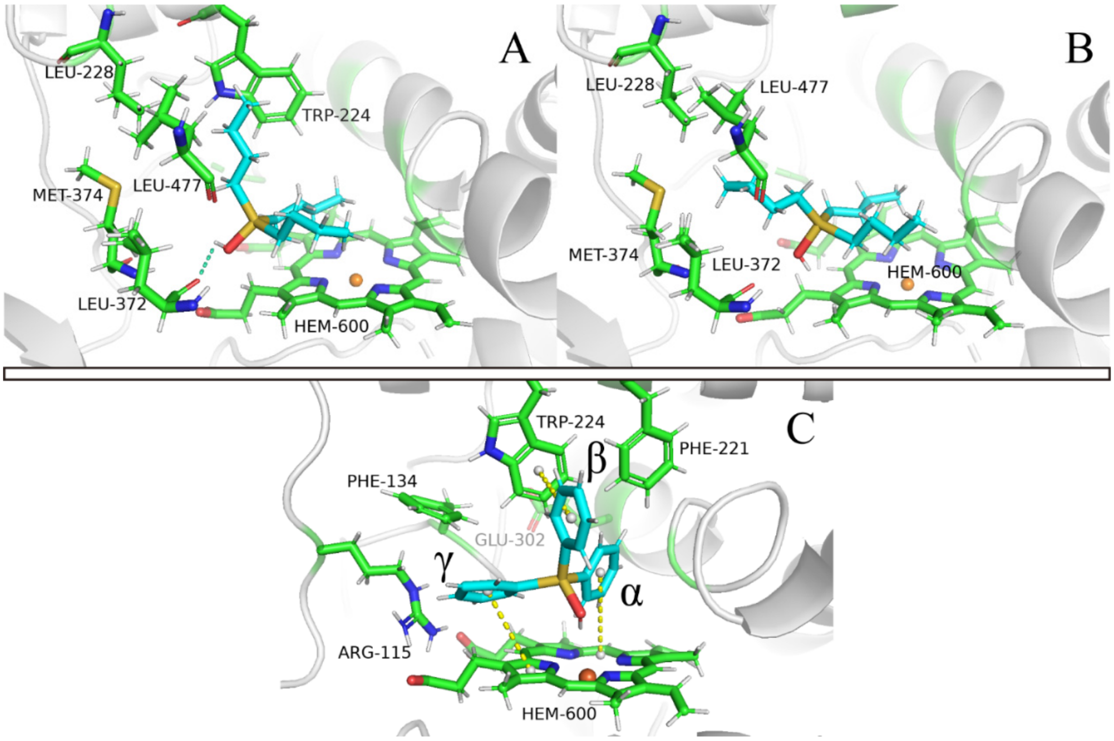

Figure S4. The binding modes of TBTOH (A)(B) and TPTOH (C) with aromatase in the 3-30 ns trajectories. The hydrogen bonds are represented by cyan dash. The π-π interactions between TPTOH and TRP-224, HEM are presented as yellow dashes. (A) The dominant binding mode of TBTOH. (B) The unstable binding mode of TBTOH. C: The binding mode of TPTOH.

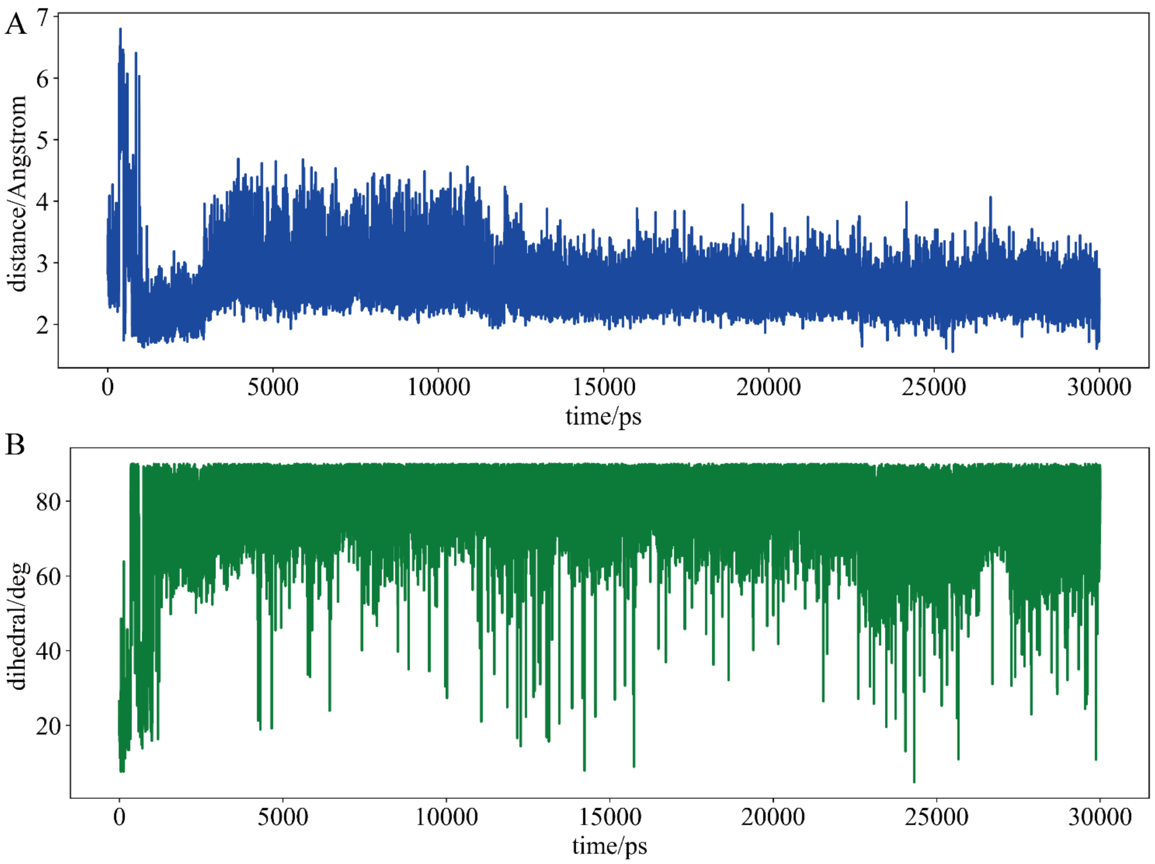

Figure S5. The π-π interaction distance (A) and dihedral (B) between TPTOH ring-α and HEM-600 as function of time during MD simulation.

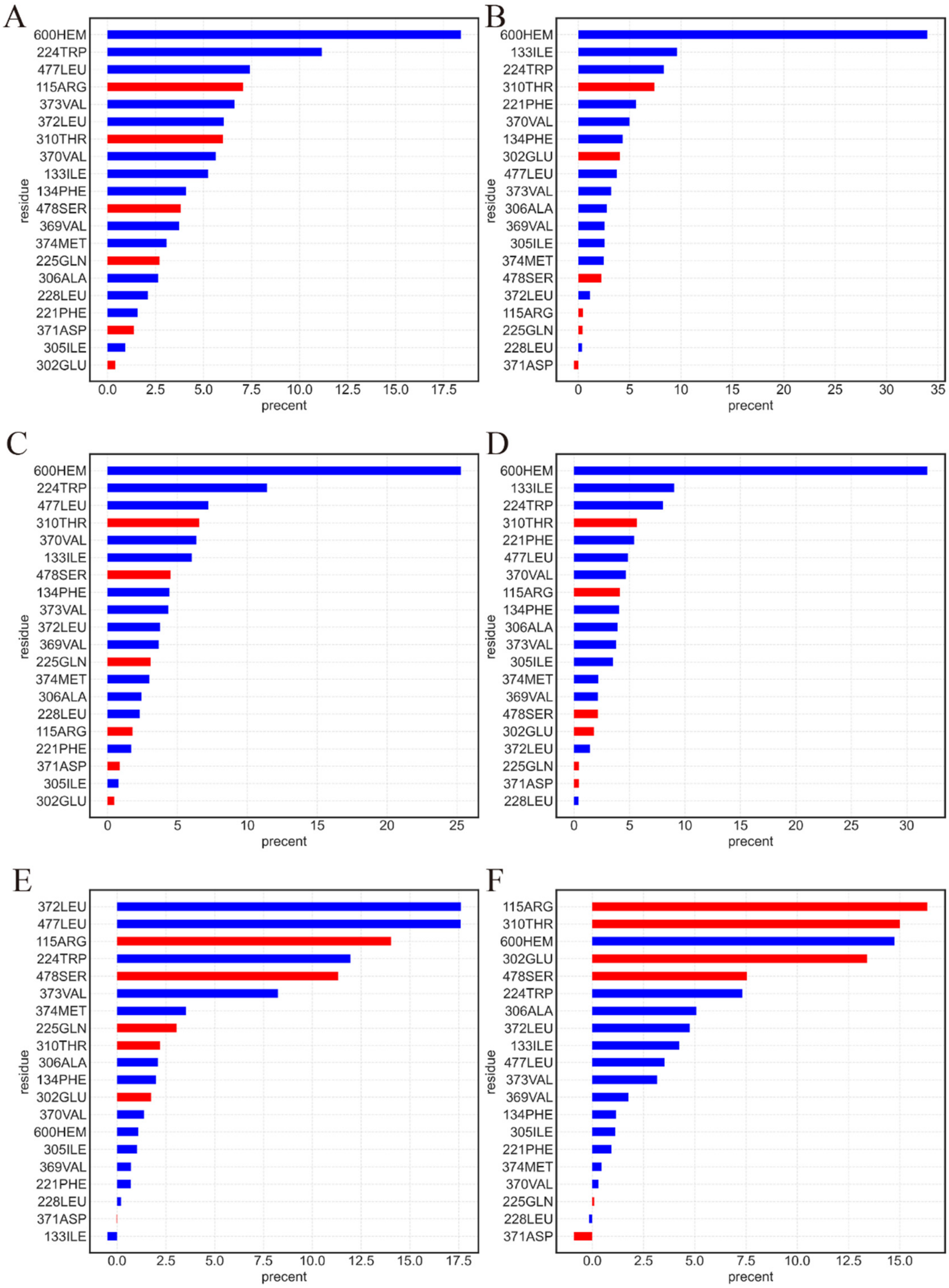

Figure S6. The contribution of each binding sites residues to the main components of ΔG_{bind}: ΔE_{gas} (A-B), van der Waals contribution (C-D) and $\Delta\Delta G_{PB}$ (E-F). (A, C, E) TBTOH. (B, D, F) TPTOH.

Table S1. The clustering result of MD trajectory. The number of frames in each cluster was shown in the table below. The cluster 1 to 10 and 13 to 17 correspond to binding mode1 of TBTOH, and cluster 11 and 12 correspond to mode2.

Cluster ID	TBTOH	TPTOH
1	838	59
2	2	3
3	2	407
4	4	7
5	218	110
6	55	37
7	185	24
8	48	1125
9	7	384
10	536	503
11	5	42
12	105	
13	155	
14	228	
15	145	
16	133	
17	35	

Table S2. The total SAPT interaction energy between TPTOH and TRP-224 or PHE-221 and its electrostatics, exchange, induction and dispersion components (kJ/mol).

Residue	Total	Electrostatics	Exchange	Induction	Dispersion
TRP-224	-13.949	-6.104	24.660	-2.424	-30.081
PHE-221	-12.247	-9.373	20.977	-2.644	-21.207

Table S3. The mean value of the short-range organotin-water interaction energy and its Coulomb and van der Waals components (kJ/mol). ΔE_{total}=ΔE_{total}+ΔE_{water}. ΔE_{water} is the sum of coulomb and van der Waals components of the short-range organotin-water interaction energy.

Ligand	Coulomb	van der Waals	ΔE_{gas}	ΔE_{total}
TBTOH	-39.332	-2.234	-202.424	-243.990
TPTOH	-43.452	-7.735	-196.965	-248.152

Definition of Bond-Forming Probability

To quantify the stability of hydrogen bonds in terms of structural information, we defined the bond-forming probability using the following equation:

$$P = \frac{N_{hbond}}{N_{tot}}$$

N_{hbond} is the number of frames in which the hydrogen bond forms; N_{tot} is the total number of frames in a trajectory. We use a criterion that the donor-receptor distance d(H···X, X=O or N) is below 2.5 Å and the hydrogen bond angle is greater than 120° to identify the hydrogen bonding. The bond-forming probability was calculated on the 2701 frames extracted from the 3–30 ns trajectory using the uniform sampling method with a sampling interval of 10 ps.
